# Performance of Nano- and Microcalcium Carbonate in Uncrosslinked Natural Rubber Composites: New Results of Structure–Properties Relationship

**DOI:** 10.3390/polym12092002

**Published:** 2020-09-03

**Authors:** Nantikan Phuhiangpa, Worachai Ponloa, Saree Phongphanphanee, Wirasak Smitthipong

**Affiliations:** 1 Specialized Center of Rubber and Polymer Materials in Agriculture and Industry (RPM), Department of Materials Science, Faculty of Science, Kasetsart University, Bangkok 10900, Thailand; wernnantikan@gmail.com (N.P.); worachai.p@ku.th (W.P.); fscisrph@ku.ac.th (S.P.); 2 Office of Research Integration on Target-Based Natural Rubber, National Research Council of Thailand (NRCT), Bangkok 10900, Thailand; 3 Office of Natural Rubber Research Program, Thailand Science Research and Innovation (TSRI), Bangkok 10400, Thailand

**Keywords:** nanocalcium carbonate, natural rubber nanocomposite, Mullins effect, stress relaxation, Payne effect

## Abstract

Calcium carbonate (CaCO_3_) is one of the most important inorganic powders and is widely used as filler in order to reduce costs in the rubber industry. Nanocalcium carbonate reduces costs and acts as a semireinforcing filler that improves the mechanical properties of rubber composites. The objective of this study was to investigate the effect of nano-CaCO_3_ (NCC) and micro-CaCO_3_ (MCC) on the properties of natural rubber composites, in particular, new results of structure–properties relationship. The effects of NCC/MCC on the properties of rubber composites, such as Mooney viscosity, bound rubber, Mullins effect, and Payne effect, were investigated. The result of the Mullins effect of rubber composites filled with NCC was in good agreement with the results of Mooney viscosity and bound rubber, with higher Mooney viscosity and bound rubber leading to higher stress to pull the rubber composites. The Payne effect showed that the value of different storage moduli (ΔG’) of rubber composites filled with 25 parts per hundred rubber (phr) NCC was the lowest due to weaker filler network, while the rubber supplemented with 100 phr NCC had more significant ΔG’ values with increase in strain. The results of rubber composites filled with MCC showed the same tendency as those of rubber composites filled with NCC. However, the effect of specific surface area of NCC on the properties of rubber composites was more pronounced than those of rubber composites filled with MCC. Finite element analysis of the mechanical property of rubber composites was in good agreement with the result from the experiment. The master curves of time–temperature superposition presented lower free volume in the composites for higher loading of filler, which would require more relaxation time of rubber molecules. This type of nanocalcium carbonate material can be applied to tailor the properties and processability of rubber products.

## 1. Introduction

Natural rubber (NR) is a major agricultural product that is widely used in the rubber industry for the production of tile floor, tires, gloves, pillows and mattresses, medical products, etc. NR can be obtained from *Hevea brasiliensis*, which consists of rubber (*cis*-1,4-polyisoprene) and nonrubber components (proteins, phospholipids, sugars, salts, etc.) [1,2,3]. To date, there is no other synthetic material that can replace natural rubber from plants [4]. NR is often reinforced by incorporation of filler to improve its mechanical properties, namely, modulus, hardness, tensile strength, abrasion resistance, and tear resistance [5].

Recently, fillers have been widely used in the rubber industry for many purposes, such as improvement of mechanical properties, efficient production, and reduction in cost of rubber products [6,7,8,9,10,11]. Generally, there are three main groups of fillers: reinforcing fillers, semireinforcing fillers, and nonreinforcing fillers. The efficiency of the reinforcing filler depends on several factors, such as particle size, surface area, and the shape of filler [12,13,14]. Among commercial fillers, carbon black and silica (SiO_2_) are the most important reinforcing fillers. They are added to improve the mechanical properties of rubber compounds. However, there are some fillers that can be used as non- or semireinforcing fillers (such as calcium carbonate, clay, talc, etc.) for either reducing cost or improving mechanical properties [15]. 

Calcium carbonate (CaCO_3_) is one of the most important inorganic powders and is widely used as filler in paints, plastics, and the rubber industry in order to reduce the material cost [16]. Calcium carbonate is considered as a filler for rubbers. Its surface property, controlled shape, and small particle size affect the processing and properties of rubber composites. Calcium carbonate can be coated by hydrophobic molecules in order to better interact with hydrophobic rubber [17]. Based on modern technology, the particle size of CaCO_3_ can be reduced to the nanoscale [18]. Nanocalcium carbonate (NCC) can be added to rubber, and the mechanical properties of the nanocomposites increases with increasing amount of NCC [19]. Moreover, the dynamic characteristics of NCC added to crosslinked NR has been studied, and the results showed that the shape of NCC affects the Mullins and Payne effects of the rubber composite [20]. NCC can be used as fillers not only to decrease the cost of materials but also to increase the mechanical properties of the crosslinked rubber composites [21]. 

However, there is no known research on the structure–properties relationship of nanocalcium carbonate in uncrosslinked NR nanocomposites compared to microcalcium carbonate in uncrosslinked NR composites, which can be beneficial for potential applications of nanocalcium carbonate in the rubber industry. Thus, the main aim of this work was to investigate the effect of both nano-CaCO_3_ (NCC) and micro-CaCO_3_ (MCC) at a wide range of filler loading on the properties of uncrosslinked rubber composites, in particular, new results of structure–properties relationship.

## 2. Materials and Methods 

### 2.1. Materials 

The main material were nanocalcium carbonate (particle size around 80 nm, Sand and Soil Industry Co., Ltd., Bangkok, Thailand), microcalcium carbonate (particle size around 1.30 µm, Sand and Soil Industry Co., Ltd., Bangkok, Thailand), natural rubber (STR 5L, Rubber Authority of Thailand, Bangkok, Thailand), and toluene (AR grade, RCI Labscan Limited, Bangkok, Thailand). 

### 2.2. Preparation of Composites

Natural rubber, in total weight of rubber for 500 g of each formulation, was first added into a two-roll mill and masticated at 70 °C for 5 min. After that, the amount of either NCC or MCC was added into the NR according to Table 1 and then mixed together at 70 °C for 15 min. Finally, all samples were cut into the form of testing sheets.

### 2.3. Characterizations

For scanning electron microscopy analysis (SEM; FEI, Quanta 450 FEI, Eindhoven, Netherlands), rubber samples were cut into small pieces and coated with gold in a sputter coater (Polaron Range SC7620, Quorum Technologies Ltd., Kent, UK) for the morphology analysis. 

The Mooney viscosity of rubber composites was determined by a Mooney viscometer (viscTECH+, Techpro, Columbia city, IN, USA); the weight of the sample was 12 g. The Mooney viscometer consists of a rotating disc imbedded in a rubber specimen contained within a sealed, pressurized, and heated cavity. The rubber sample was heated at 100 °C for 1 min before starting the motor. After that, the rubber sample was continuously measured for 4 min using the torque required to keep the rotor rotating at a constant rate as a function of time for reading the Mooney viscosity, which was recorded as torque in newton meter (Nm). 

Bound rubber was considered a quantitative measure of the filler surface activity and the rubber–filler interaction. Bound rubber was determined by immersing 1 g of rubber composite in 100 mL of toluene solvent at room temperature for 7 days. After dissolution, a piece of rubber that is insoluble in toluene was filtered, weighed, and then calculated with respect to the original sample weight [22]. 

The Mullins effect of the rubber composites was determined by a universal testing machine (AGS-X,20 N, Shimadzu, Tokyo, Japan); the rubber sample was cut into dumbbell shape. The condition of test in the strain axis was varied 1–120% in cycle mode, and then pulling–releasing of the sample was carried out three times. 

Stress relaxation of the rubber composites was determined by dynamic mechanical analysis (DMA1, Mettler Toledo, Columbus, OH, USA). The rubber was cut into samples of 2 mm width, 4 mm length, and 1.5 mm thickness and tested at a temperature of 30 °C with strain range of 10% for 600 s. A curve measured on a DMA-type module using segments of the stress relaxation type can be displayed in a stress relaxation diagram as a stress–time curve. The stress–time curve presented the actual force measurement signal and the cross-sectional area of the sample as a function of time.

The Payne effect of either NCC- or MCC-supplemented rubber composites was analyzed using a rubber processing analyzer (RPA 2000, Alpha Technologies, Hudson, OH, USA) under the following conditions: temperature 60 °C, frequency 1 Hz, strain range 1–100%. The value of different storage moduli (ΔG’) means G’_max_–G’_min_. 

Viscoelastic properties of the rubber composites were determined by dynamic mechanical analysis (DMA1, Mettler Toledo, Columbus, OH, USA) based on Williams–Landel–Ferry (WLF) analysis; the rubber composite was cut into samples of 2 mm width, 4 mm length, and 1.5 mm thickness and tested at a temperature range of −80 to 50 °C and frequency of 1–100 Hz. The time–temperature superposition principle was used to establish a master curve of the storage modulus (E’) as a function of reduced frequency at a reference temperature *T_ref_* of 298 K [23]. Shift factors (*a_T_*) for the establishment of master curves were determined according to the WLF equation, where *C*_1_ and *C*_2_ are constants depending on the nature of the elastomer and the reference temperature [24].

### 2.4. Finite Element Analysis

The material parameters for the rubber composites were determined by incompressible isotropic hyperelastic strain energy models. In this work, we applied the reduced polynomial form of strain energy potential from order 3 (or Yeoh model) to 6. To get the parameters of the models, the finite element method (FEM) and curve fitting analysis were carried out using ABAQUS on uniaxial tension of the rubber composites. For the hyperelastic materials, the strain energy function of incompressible is the function of strain invariants, *I* [25]:

(1) $$W=W\left(I_{1},I_{2},I_{3}\right)$$

The strain invariants can be written as follows:

(2) $$I_{1}=\lambda_{1}^{2}+\lambda_{2}^{2}+\lambda_{3}^{2}$$

(3) $$I_{2}=\lambda_{1}^{2}\lambda_{2}^{2}+\lambda_{2}^{2}\lambda_{3}^{2}+\lambda_{3}^{2}\lambda_{1}^{2}$$

(4) $$I_{3}=\lambda_{1}^{2}\lambda_{2}^{2}\lambda_{3}^{2}$$

where $\lambda_{1}$, $\lambda_{2}$, and $\lambda_{3}$ are the principal stretches. The strain energy function of reduced polynomial for incompressible rubber can be written as follows:

(5) $$W=\overset{N}{\underset{i=1}{\sum }}C_{i0}{\left(I_{1}-3\right)}^{i}$$

where $C_{i0}$ is the temperature-dependent parameter for materials. The parameter $C_{10}$ is related to the initial shear modulus, $\mu_{0}$, by $2C_{10}=\mu_{0}$.

## 3. Results and Discussion

### 3.1. Morphological Properties

**Figure 1:**
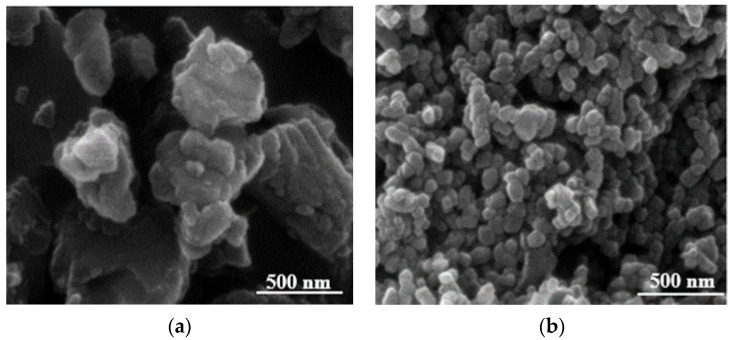
The SEM images of (**a**) micro-CaCO_3_ (MCC) and (**b**) nano-CaCO_3_ (NCC) in spherical shape.

The SEM images (Figure 1) of MCC and NCC showed spherical shape for both types of calcium carbonates. These results are in good agreement with previous works [20,26]. It was observed that the MCC particles had larger particles compared to NCC at the same magnification. Regarding the supplier certificate, MCC had around 1.30 µm particle size compared to around 80 nm for NCC. Besides the smaller primary particle size of NCC, it could be seen that the nanoparticles were grouped together into aggregates. As each aggregate still had attraction forces from the specific surface area, those groups could bundle further to form larger agglomerates. Nevertheless, the primary particle size of NCC was, on average, an order of magnitude smaller than MCC, even though both had the same spherical shape.

**Figure 2:**
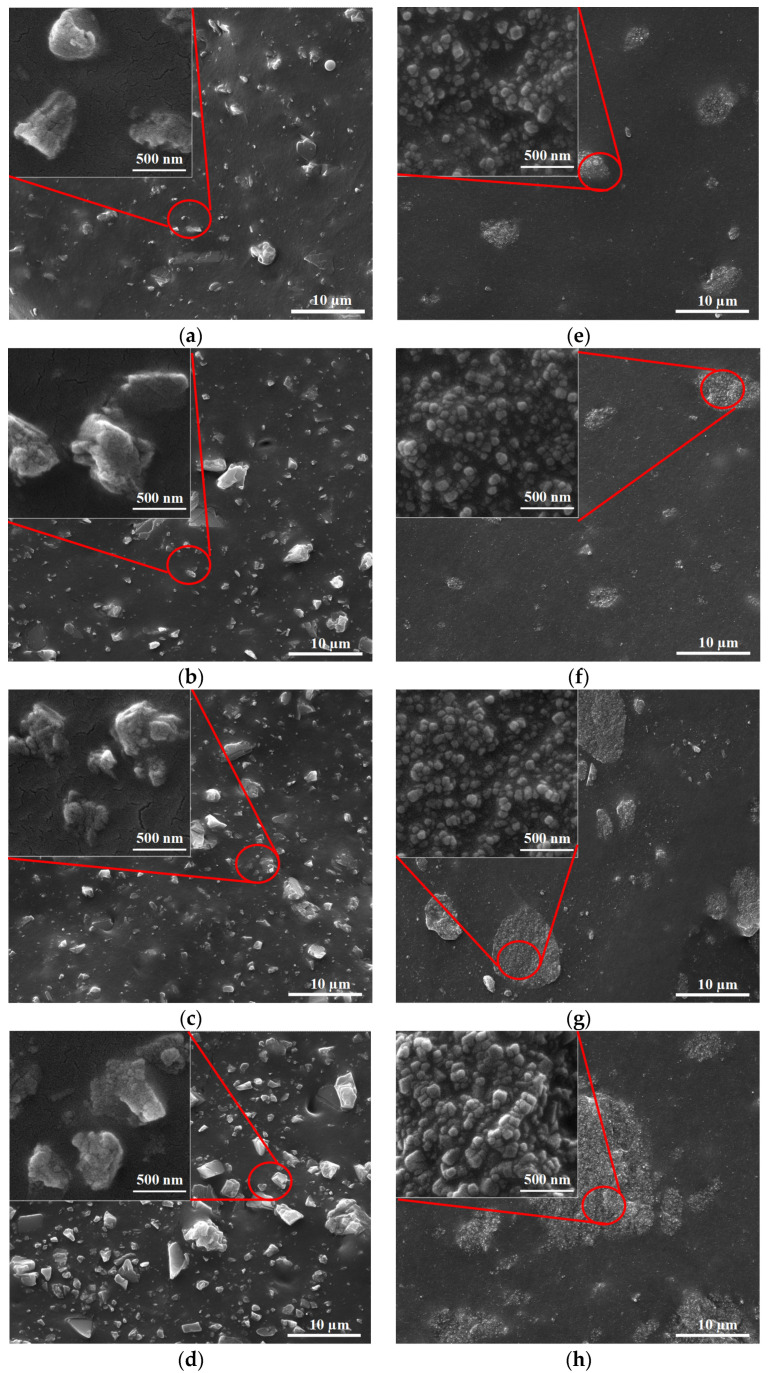
SEM images of rubber composites: (**a**) NR/MCC 25 phr, (**b**) NR/MCC 50 phr, (**c**) NR/MCC 75 phr, (**d**) NR/MCC 100 phr, (**e**) NR/NCC 25 phr, (**f**) NR/NCC 50 phr, (**g**) NR/NCC 75 phr, and (**h**) NR/NCC 100 phr.

When analyzing the natural rubber with MCC, it was found that the distribution of particles was proportional to the amount of filler (Figure 2). When NCC was added to the rubber, there were some parts of the NCC that were evenly distributed and some agglomerated together according to the amount of filler. When more fillers were added, the agglomerate became larger. Therefore, there was a filler–filler interaction based on the specific surface area of nanocalcium carbonate, in particular, resulting in filler agglomeration of NCC (Figure 2).

### 3.2. Physical Properties

**Figure 3:**
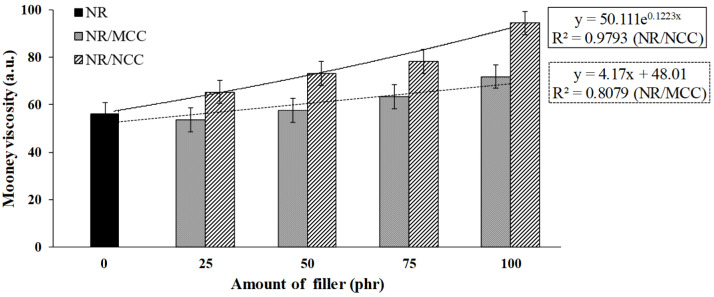
Mooney viscosity and linear relationship of natural rubber with either MCC or NCC at 25, 50, 75, and 100 phr.

The Mooney viscosity test was performed in order to study the relationship between the macromolecular structure of NR filled with calcium carbonate and viscosity. Figure 3 shows the Mooney viscosity of natural rubber with either MCC or NCC. It was found that the Mooney viscosity of the rubber composites increased with increasing amount of either MCC or NCC into natural rubber. Because MCC and NCC are fillers that have solid particles, when mixed with natural rubber, the fillers can increase the Mooney viscosity of the rubber composite. When a filler (rigid material) is mixed into a rubber (soft material), the filler blocks the flow of rubber, which is called a “hydrodynamic effect” [27]. This causes the increase in Mooney viscosity of the rubber composites. Comparing MCC and NCC, the nanosized filler NCC had a much higher ratio of surface area per volume than the microsized filler MCC. This would probably cause higher movement restriction in the rubber composites. Therefore, more torques were required in the Mooney viscosity test of NCC blended rubber.

The Mooney viscosity of the samples was almost stable when MCC at both 25 and 50 phr was added into NR. However, when the MCC was more than 50 phr, the Mooney viscosity of the rubber composites increased. In contrast, the Mooney viscosity of NR with NCC exponentially increased when the NCC was only at 25 phr, indicating that the smaller filler was able to interact with rubber more effectively (Figure 3). This result is in good agreement with a previous study, where the viscosity of epoxy composites supplemented with either graphene nanoplatelets or graphite was found to exponentially increase with the loading of filler [28]. 

**Figure 4:**
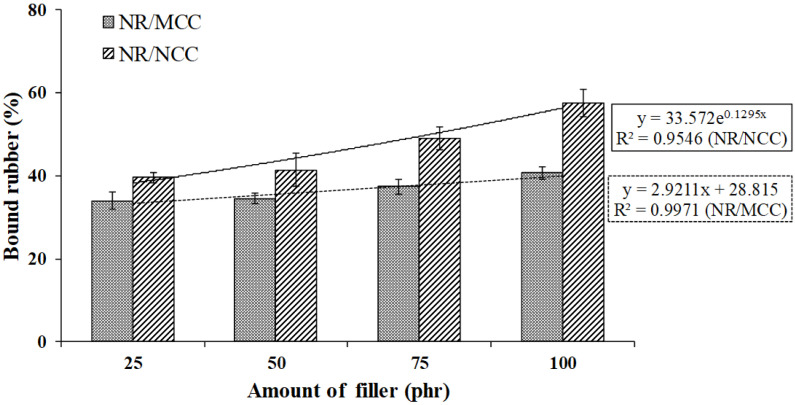
Bound rubber for natural rubber (NR) with various sizes (MCC vs. NCC) and amounts of filler (25, 50, 75, and 100 phr).

We studied the interaction of rubber–filler using bound rubber, which was measured by extraction of the rubber-free chain in the composite with toluene at room temperature [14]. The percentages of bound rubber in all samples are summarized in Figure 4. The bound rubber of NR/MCC linearly increased with increasing MCC amount, while the bound rubber of NR/NCC exponentially increased with increasing NCC amount, certainly due to the enhancement of NR and nanosized filler interaction. This result is in good agreement with the result of Mooney viscosity.

### 3.3. Mechanical Properties

**Figure 5:**
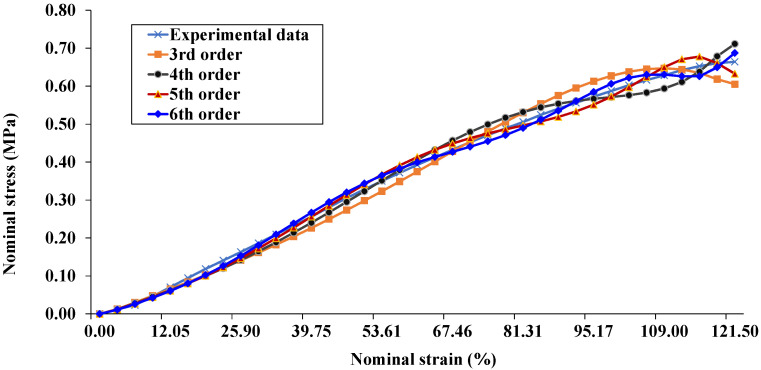
The stress–strain relationship from experiment data and hyperelastic modelling, 3rd to 6th order reduced polynomial.

**Figure 6:**
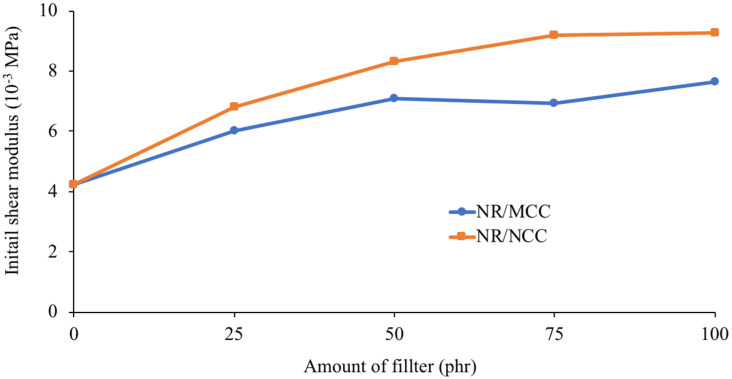
The initial shear modulus, $\mu_{0}$, at different loading of MCC and NCC.

Figure 5 demonstrates the stress–strain relationship from the experimental data and hyperelastic models of the NR system. For the reduced polynomial model used in this work, the 6th order is the best-fitting curve with experimental data. Table 2 shows the parameters from the fitting results of 6th order, and $C_{10}$ is related to the initial shear modulus by $\mu_{0}=2C_{10}$, which are also shown in the same table. The relationships of the initial shear modulus of NR composites are illustrated in Figure 6. It can be seen that the initial shear modulus of NR/NCC was higher than that of NR/MCC at a given loading of filler. This result is in good agreement with the results of Mooney viscosity and bound rubber.

**Figure 7:**
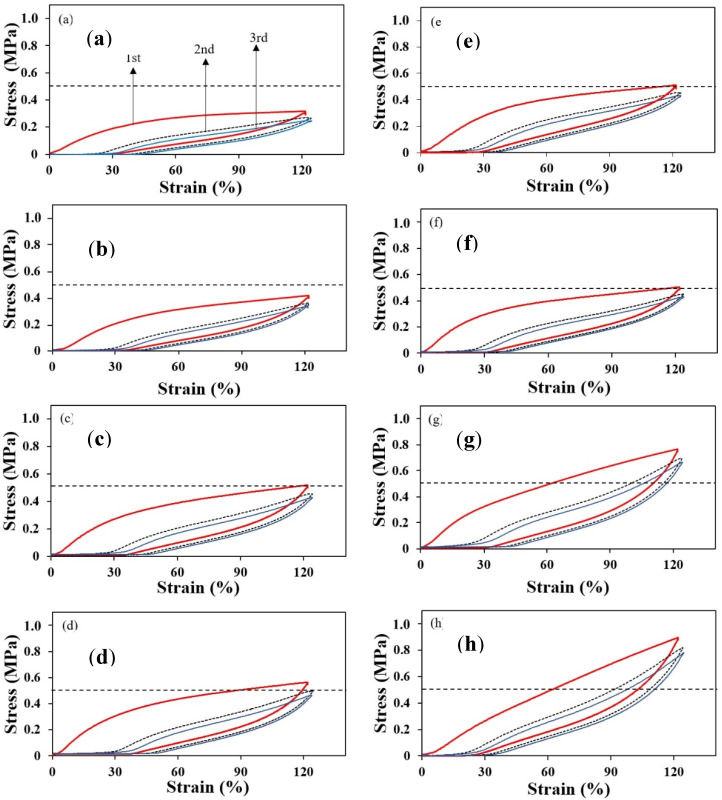
Mullins effect of NR/MCC (**left**) and NR/NCC (**right**) composites when the amount of either MCC or NCC were at (**a**,**e**) 25 phr, (**b**,**f**) 50 phr, (**c**,**g**) 75 phr, and (**d**,**h**) 100 phr.

The Mullins effect is a phenomenon observed in rubber composite where the stress–strain equilibrium causes a strain-induced softening character between rubber and filler [29]. Forces are repeatedly applied to a rubber composite, which leads to weakening of the sample. In this study, the Mullins effect was analyzed by stress–strain curves (three loops of go-return curves) for natural rubber with addition of either MCC or NCC at 25, 50, 75, and 100 phr (Figure 7). We found that the Mullins effect of NR/MCC was stable below 50 phr of MCC; beyond this concentration, the Mullins effect of NR/MCC was more pronounced. The shape of Mullins effect of NR/NCC also changed above 50 phr of NCC. Meanwhile, NR/NCC had a higher Mullins effect than NR/MCC at a given concentration of filler, indicating that the high agglomeration of nanoparticle (NCC) contributes to the high Mullins effect. This filler agglomeration can hinder the mobility of the macromolecular chains when pulling; thus, higher stress concentration at the localized spot can occur [30]. The microsized CaCO_3_ could easily slip between macromolecular chains in the NR matrix, so it could decrease the stress softness. For all the samples, the stress–strain curves of the second and third loops were always less pronounced in Mullins effect compared to the first loop. 

Based on the results of the Mullins effect, we were also interested to investigate the stress relaxation of rubber molecules filled with either MCC or NCC, which can occur through viscoelastic flow or slippage of entanglements loosening the network of rubber chains or may arise from scission of the rubber chains supporting the stress. Stress relaxation of a composite occurs when a constant stress creates physical and/or chemical changes between the rubber molecule and the filler, thus reducing the force that the composite exerts over a certain period of time. Then, the relaxation of the rubber chains and fillers can be presented. If the changes that occur are generally chemical reactions, the effects tend to be long-term and irreversible [31,32].

**Figure 8:**
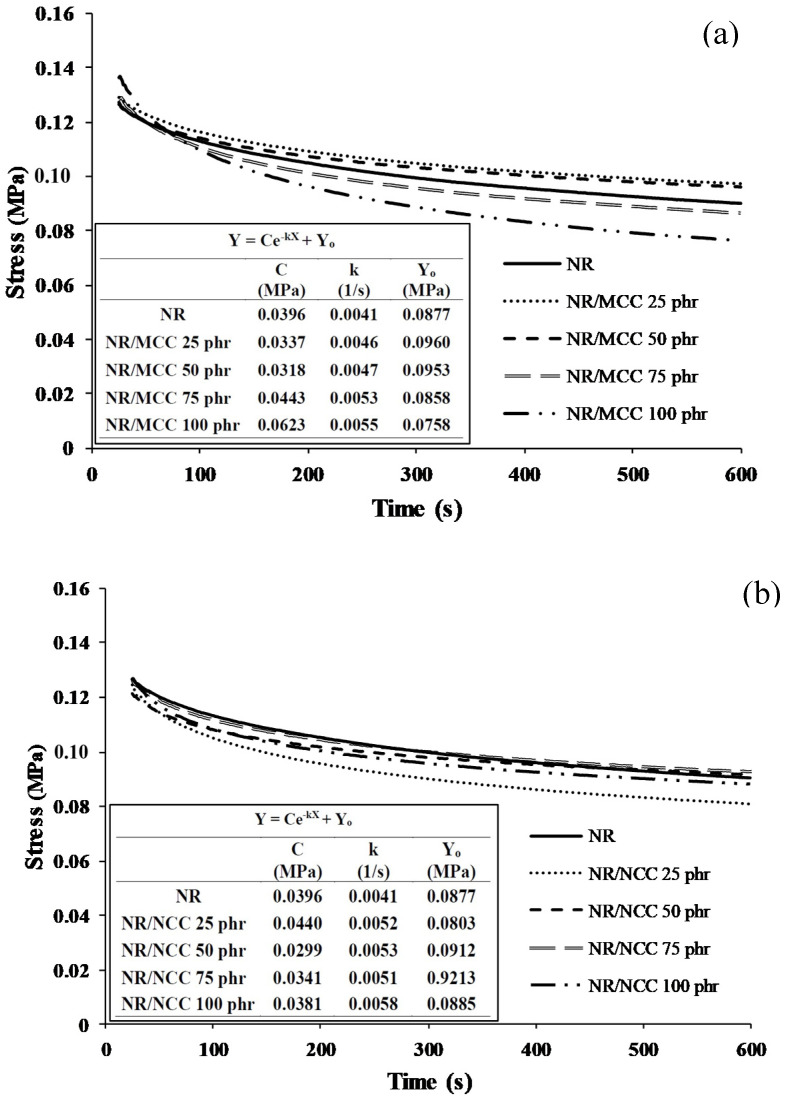
Stress relaxation curves of samples: (**a**) pure NR and NR with MCC at 0, 25, 50, 75, and 100 phr, (**b**) pure NR and NR with NCC at 0, 25, 50, 75, and 100 phr.

Figure 8a shows the stress relaxation curves of pure NR and NR/MCC composites, and Figure 8b shows the stress relaxation curves of pure NR and NR/NCC composites. We plotted a fitted curve and then extracted the values in order to form a negative exponential equation Y = Ce−^kX^ + Yo, where C is the difference value between initial stress and final stress, k is the rate of stress relaxation, and Yo is the final stress at time 0. We found that the rates of stress relaxation (k) for both types of filler (MCC and NCC) increased with increasing filler loading. This results is in good agreement with a previous study of organically modified montmorillonite-filled natural rubber/nitrile rubber nanocomposites, where the rate of stress relaxation was also found to increase with increasing filler loading [33] due to the increase in interaction, thus being more pronounced in entropy. 

Focusing on the filler–filler interaction and particularly the effect of calcium carbonate size on composite properties, next, we examined properties of the composites in shear mode of the rubber processing analyzer. The Payne effect is a feature of the stress–strain behavior, which is related to the shape change caused by strain in the rubber with fillers [34]. This phenomenon is related to the storage modulus (G’) and loss modulus (G”) in shear deformation conditions. The reason for this phenomenon is the formation of a network formed by filler–filler interaction in the composites at low strain. In real life, this means the energy loss (tan delta) and the efforts are to minimize this Payne effect in filled rubber. 

**Figure 9:**
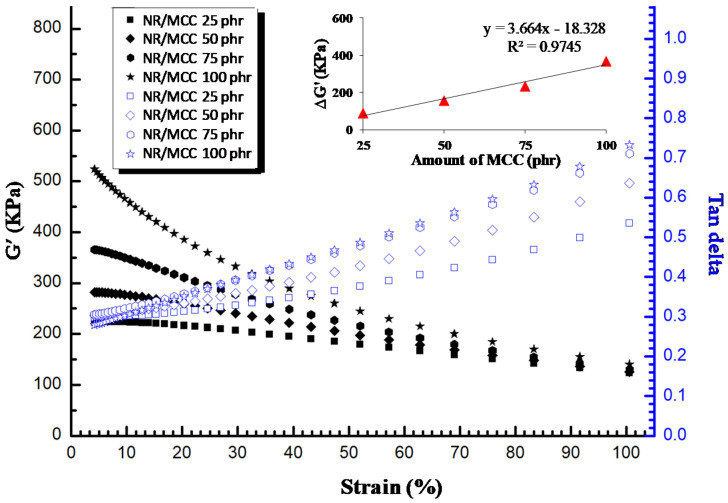
Payne effect (**left**; solid) and damping factor (tan delta, **right**; open) as a function of strain for NR with MCC particles at 25–100 phr.

**Figure 10:**
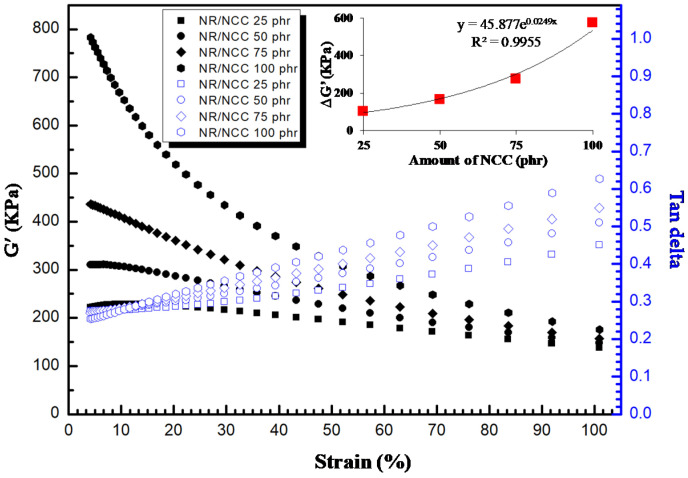
Payne effect (**left**; solid) and damping factor (tan delta, **right**; open) as a function of strain for NR with NCC particles at 25–100 phr.

Figure 9 shows the Payne effect of NR/MCC composites. We found that NR/MCC at 25 phr of MCC possessed the lowest value of different storage moduli (ΔG’), certainly due to the weaker filler network, which resulted in the largest interaggregate distance in the rubber matrix. Meanwhile, NR/MCC at 100 phr of MCC possessed the highest value of different storage moduli (ΔG’). This Payne effect can be applied to explain the destruction–reformation of filler–filler networks and adsorption–desorption of rubber chains at the filler interface of the rubber composite [21]. The fact is that upon loading of MCC, the interaggregate distances become smaller with increasing filler content, and the probability of the formation of a filler network therefore increases. The damping peak (tan delta) of the composites with MCC continuously increased with increasing strain, with higher value of ΔG’ possessing higher damping peak or hysteresis for the same sample system (Figure 9). Therefore, the hysteresis resulted from the breakdown of the filler network, and the straining disruption could dissipate energy. Concerning the NR/NCC composites (Figure 10), their values of ΔG’ were higher than those of NR/MCC composites. Moreover, the values of ΔG’ for NR/NCC composites exponentially increased with increasing filler content, whereas the NR/MCC composites possessed a linear increase of ΔG’ with increasing filler content. This can be explained by the pronounced effect of the filler–filler network of NCC. Interestingly, NR/NCC composites had more filler–filler interaction or agglomeration than NR/MCC composites at a given loading of filler, while the tan delta of NR/NCC composites was lower than that of NR/MCC composites. This might have come from the synergy effect between bound rubber and the Payne effect. NR/NCC composites had higher bound rubber or network of rubber–filler interaction than NR/MCC composites, so the NR/NCC composites possessed lower heat build-up within the material. Higher rubber networks possess lower dissipation of energy or tan delta [2]. 

Then, we also estimated the reinforcement efficiency of the filler in NR matrix (*α_f_*) as a modified equation below [35]:

(6) $$\alpha_{f}\;=\;\frac{\frac{M_{f}}{M_{g}}\;-\;1}{w}$$

where *M_f_* and *M_g_* are the Mooney viscosity for the filled composite and pure gum, respectively, and *W* is the mass fraction of the filler in the composites. This parameter (*α_f_*) is a measurement of the filler activity in the polymer matrix, so higher numerical value of *α_f_* indicates higher polymer–filler interactions [34].

**Figure 11:**
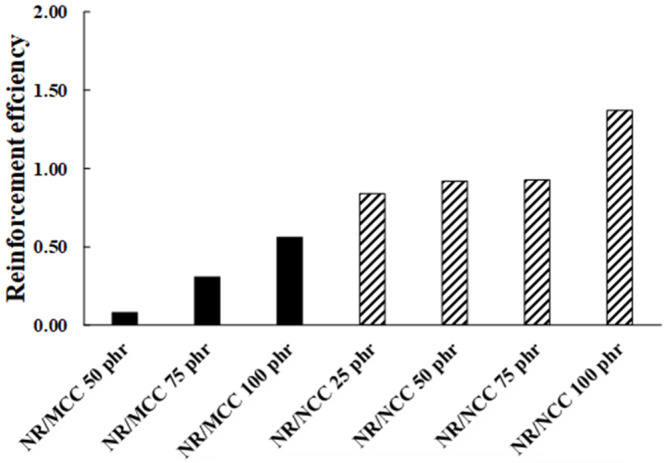
Filler reinforcing efficiency in different NR composites.

We found that the reinforcement efficiency of NR/NCC composites was higher than that of NR/MCC composites (Figure 11). This result is in good agreement with the result of the Payne effect. There was no reinforcement efficiency of NR/MCC at 25 phr. However, we applied this equation for the Mooney viscosity of green rubber composites compared to the original equation, which uses the torque from rheometer of crosslinked composites [35].

Master curves (time–temperature superposition) was determined by dynamic mechanical analysis, which represents the storage modulus of rubber samples as a function of reduced frequency. Table 3 shows the mean values of the constants *C*_1_ and *C*_2_ for universal, Ferry, filled composites and pure rubber, respectively. These two constant values are in good agreement with the Ferry reference and previous studies [23,24,36]. All the composite samples possessed the same *C*_1_, *C*_2_, and shift factor (*a_T_*) values as those of the pure NR sample [36]. 

**Figure 12:**
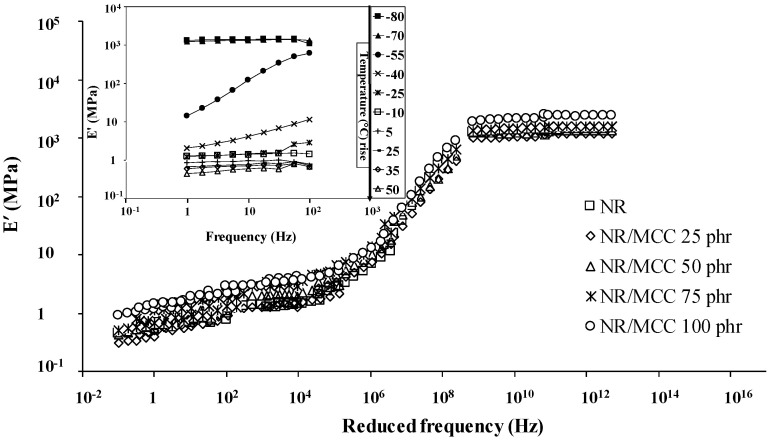
Elastic modulus (E′) versus reduced frequency master curves for pure NR and NR/MCC composites. Insert figure presents raw data of pure NR before establishing the master curve.

**Figure 13:**
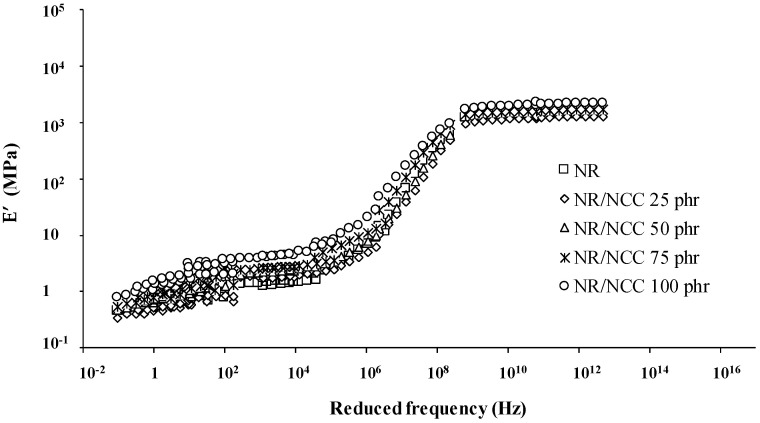
Elastic modulus (E′) versus reduced frequency master curves for pure NR and NR/NCC composites.

Both Figure 12 (NR/MCC) and Figure 13 (NR/NCC) present the master curves of rubber composites in three zones: glassy plateau at high reduced frequency, transition state, and rubbery plateau at low reduced frequency. It can be seen that all the rubbers had almost similar viscoelastic properties irrespective of whether NR was filled with MCC or NCC at different temperatures and frequencies. The viscoelastic properties of NR composites at low temperature was equivalent to those of NR composites at high frequency and vice versa. However, we also found that NR/MCC and NR/NCC at glassy state were influenced by the addition of large quantity of fillers, indicating the lower free volume in the composites for higher loading of filler. For the rubbery state, composites with higher loading of filler had higher level in this rubbery plateau, indicating more relaxation time of rubber molecules.

## 4. Conclusions

This work focused on investigating the influence of calcium carbonate morphological parameters (polymorph and different particle sizes) on the properties of uncrosslinked rubber composite, in particular, new results of structure–properties relationship. The different size of calcium carbonate has an influence on the static and dynamic mechanical properties of rubber composites corresponding to the polymorph and its particle sizes. Hence, based on the results of this study, the SEM images of MCC and NCC particles showed the same spherical shape with different sizes. Finite element analysis of the rubber composites was in good agreement with the results of experimental data. The Mullins effect of rubber composites filled with either MCC or NCC was in good agreement with the results of Mooney viscosity and bound rubber, with higher Mooney viscosity and bound rubber leading to higher stress to pull the rubber composites. The rate of stress relaxation increased with increasing filler loading for both MCC and NCC. The Payne effect showed that the value of different storage moduli (ΔG′) of rubber composites filled with 25 phr NCC was the lowest due to weaker filler network, while the NR supplemented with 100 phr NCC had more significant ΔG′ with the increase in strain. The results of rubber composites filled with MCC showed the same tendency as those of rubber composites filled with NCC. However, the effect of specific surface area of NCC on the properties of rubber composites was more pronounced than those of rubber composites filled with MCC. The master curves from WLF superposition showed similar viscoelastic properties for both NR/MCC and NR/NCC composites. This research elucidates that we can utilize the optimum amount of nanocalcium carbonate (25–100 phr depending on the product’s requirement) in order to enhance the properties and processability of natural rubber composites.

## Figures and Tables

**Table 1:** Formulations of rubber composites.

Ingredients	Parts Per Hundred of Rubber (phr) for Each Formulation								
	NR	NR/NCC	NR/NCC	NR/NCC	NR/NCC	NR/MCC	NR/MCC	NR/MCC	NR/MCC
NR	100	100	100	100	100	100	100	100	100
NCC	-	25	50	75	100	-	-	-	-
MCC	-	-	-	-	-	25	50	75	100

**Table 2:** Calibrated parameters of 6th order of reduced polynomial for the rubber composites.

	$C_{10}$ **(10^−3^)**	$C_{20}$ **(10^−7^)**	$C_{30}$ **(10^−10^)**	$C_{40}$ **(10^−14^)**	$C_{50}$ **(10^−18^)**	$C_{60}$	$\mu_{0}$ **(10^−3^)**
NR	2.12	6.59	−1.60	1.79	−0.95	0.0	4.24
NR/MCC 25 phr	3.01	0.02	−0.39	0.55	−0.32	0.0	6.01
NR/MCC 50 phr	3.55	2.71	−1.14	1.41	−0.77	0.0	7.10
NR/MCC 75 phr	3.47	4.90	−1.71	2.08	−1.13	0.0	6.94
NR/MCC 100 phr	3.83	6.10	−2.07	2.50	−1.36	0.0	7.65
NR/NCC 25 phr	3.41	6.05	−1.96	2.33	−1.25	0.0	6.82
NR/NCC 50 phr	4.15	2.96	−1.40	1.80	−1.00	0.0	8.31
NR/NCC 75 phr	4.60	3.39	−1.40	1.82	−1.03	0.0	9.19
NR/NCC 100 phr	4.63	7.64	−2.53	3.07	−1.67	0.0	9.27

**Table 3:** The constants *C*_1_ and *C*_2_ for calculating the shift factor [23,24].

Reference	*C*_1_	*C*_2_
Universal	17.44	51.60
Ferry	5.94	151.60
All NR samples	8.50	186.50
